# Eccentric Training Interventions and Team Sport Athletes

**DOI:** 10.3390/jfmk4040067

**Published:** 2019-09-27

**Authors:** Conor McNeill, C. Martyn Beaven, Daniel T. McMaster, Nicholas Gill

**Affiliations:** 1 Te Huataki Waiora School of Health, Adams Centre, The University of Waikato, 3116 Tauranga, New Zealanddmcmaste@waikato.ac.nz (D.T.M.); nicholas.gill@nzrugby.co.nz (N.G.); 2 New Zealand Rugby Union, 6011 Wellington, New Zealand

**Keywords:** eccentric, overload, training, athlete, team

## Abstract

Eccentric resistance training has been shown to improve performance outcomes in a range of populations, making it a popular choice for practitioners. Evidence suggests that neuromuscular adaptations resulting from eccentric overload (EO) and accentuated eccentric loading (AEL) methods could benefit athletic populations competing in team sports. The purpose of this review was to determine the effects of eccentric resistance training on performance qualities in trained male team sport athletes. A systematic review was conducted using electronic databases PubMed, SPORTDiscus and Web of Science in May 2019. The literature search resulted in 1402 initial articles, with 14 included in the final analysis. Variables related to strength, speed, power and change of direction ability were extracted and effect sizes were calculated with a correction for small sample size. Trivial, moderate and large effect sizes were reported for strength (−0.17 to 1.67), speed (−0.08 to 1.06), power (0.27 to 1.63) and change of direction (0.48 to 1.46) outcomes. Eccentric resistance training appears to be an effective stimulus for developing neuromuscular qualities in trained male team sport athletes. However, the range of effect sizes, testing protocols and training interventions suggest that more research is needed to better implement this type of training in athletic populations.

## 1. Introduction

There is growing evidence in the literature that suggests eccentric resistance training is an effective stimulus for enhancing physical performance [1]. During an eccentric contraction, kinetic energy is transferred and stored as elastic potential energy within the muscle tendon unit [2], which can acutely enhance force production in the subsequent concentric contraction through the stretch-shortening cycle [3,4,5]. Training methods that take advantage of the eccentric phase have been referred to in the literature as eccentric overload (EO) and accentuated eccentric loading (AEL) [6,7,8]. These terms refer to the manipulation of force and time variables during the eccentric phase of exercise through the application of relatively high force or high velocity [9,10,11]. Longitudinal eccentric training may benefit team sport athletes who are required to produce force quickly during rapid movement through favourable neuromuscular and morphological adaptations [12]. The purpose of this systematic review was to investigate the effects of EO and AEL on performance qualities in trained male team sport athletes. 

## 2. Materials and Methods

### 2.1. Search Strategy

One reviewer conducted the literature search according to the Preferred Reporting Items for Systematic Reviews and Meta-Analyses (PRISMA) guidelines for systematic reviews [13]. The electronic databases PubMed, SPORTDiscus and Web of Science were searched up until 23 May 2019. No date ranges were imposed on the individual databases. Search terms included: ‘eccentric exercise’, ‘eccentric training’, ‘eccentric contraction’, ‘strength’, ‘power’, ‘speed’, ‘velocity’, ‘force’, ‘hypertrophy’, ‘athletes’, and ‘team sports’. Boolean operators ‘AND’ and ‘OR’ were used to combine key search terms. When applicable, filters were used during the initial literature search to identify relevant articles. Full-text articles from peer-reviewed academic journals written in English were included, while articles involving animal (non-human), youth (<17 years old), and older (>44 years old) participants were excluded. 

Once the initial search had been conducted, the articles were stored in reference manager software (Zotero, version 5.0.52, Corporation for Digital Scholarship, Vienna, VA, USA). Duplicate articles were manually reviewed and merged using the included “Duplicate Items” function. From the company’s website, “Zotero assesses records for duplicates based on the title, DOI, and ISBN fields to determine duplicates. If these fields match (or are absent), Zotero also compares the years of publication (if they are within a year of each other) and author/creator lists (if at least one author last name plus first initial matches) to determine duplicates” (www.zotero.org). The titles and abstracts of the remaining records were then screened. Articles not meeting the inclusion/exclusion criteria were removed, and the remaining records were assessed. Those full-text studies meeting the eligibility criteria were then assessed for inclusion in the review. An additional search was carried out using the reference lists of articles; those records identified through the additional search were then subjected to the same systematic process. Finally, all of the studies deemed to meet the criteria were assessed for methodological quality and included in the review. 

### 2.2. Eligibility Criteria

Studies meeting the following inclusion criteria were included in the review:

- Participants were healthy, competitive, male team sport athletes above the recreational level (i.e., professional, national, elite) and were between 17 and 35 years of age.
- The sports included in the review following the screening process were basketball, soccer, handball, and rugby union.
- Studies investigated the effects of longitudinal (≥three weeks) EO training interventions. Eccentric training load (volume, intensity) needed to be quantified.
- Data on at least one of the following outcome measures were reported: strength (e.g., 1RM, maximal voluntary contraction, peak torque), maximum sprint times (e.g., 10 m, 20 m, 40 m sprint), power (e.g., jump height, rate of force development), and change of direction (e.g., T-test, cutting).

Studies with the following exclusion criteria were not included in the review:

- Participants were individual sport athletes (i.e., skiing, cycling, running) or untrained (students or with less than six months training experience). Studies not listing the training experience/sport status of participants were also excluded.
- Studies investigating male and female athletes were excluded if the results were not reported separately.
- The training intervention included injured participants.
- Supplements or ergogenic aids were used in the intervention.

### 2.3. Study Selection

**Figure 1:**
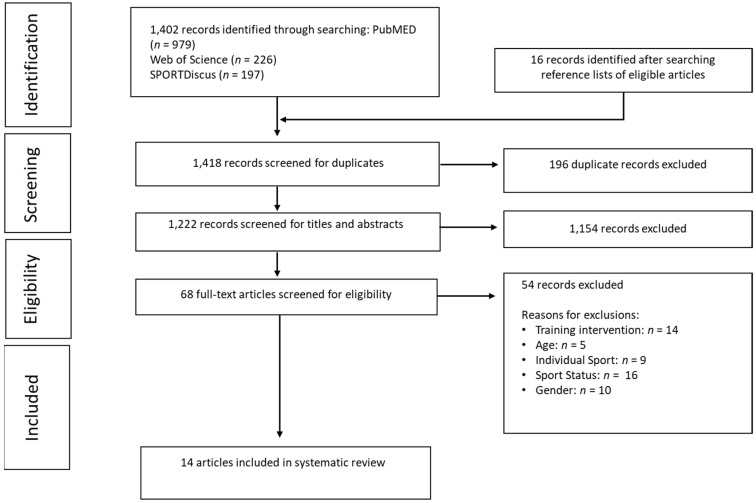
Flow chart of the literature search process using the Preferred Reporting Items for Systematic Reviews and Meta-Analyses (PRISMA) guidelines.

The eligibility assessment was performed in an unblinded manner by a single reviewer. The study selection process used is visually represented by Figure 1. Those studies identified through the initial search outlined above or identified through reference lists were then screened for eligibility criteria. If there was uncertainty about whether a study met the standard for inclusion, an additional reviewer (C. Martyn Beaven) was consulted and an agreement was reached (*n* = 5).

### 2.4. Analysis of Results 

The remaining 14 articles were evaluated using a 10 item scale designed for exercise training studies [14]. The goal of the scale was to assess the quality of strength and conditioning interventions, which might otherwise score poorly in assessments designed for healthcare research and interventions. This scale includes a 10 item scale (range 0 to 20) designed for rating the methodological quality of exercise training studies (Table 1). Two authors conducted the quality assessment independently; any discrepancies between the scores were discussed and a consensus was reached (*n* = 8). The score for each criterion was as follows: 0 = “clearly no”, 1 = “maybe”, and 2 = “clearly yes”.

The items included:

1. Inclusion criteria were clearly stated;
2. Subjects were randomly allocated to groups;
3. Intervention was clearly defined;
4. Groups were tested for similarity at baseline;
5. Use of a control group;
6. Outcome variables were clearly defined;
7. Assessments were practically useful;
8. Duration of intervention was practically useful;
9. Between-group statistical analysis was appropriate;
10. Point measures of variability.

One reviewer created a data extraction form based on several existing literature reviews [1,6,15,16] and variables of interest related to the research questions. This extraction form was created using Microsoft Excel 2016 (Microsoft Corporation, Redmond, WA, USA). The reviewer then manually extracted data from each study for the following physical qualities: strength, speed, power and change of direction. Where possible the mean, standard deviation, percent difference and effect size statistic were calculated. If the relevant information (sample size, standard deviation, change in means) was not available, then the authors’ reported values were used. Effect size was calculated for each treatment to determine the magnitude of change in the outcome variable using the mean difference (Mdiff), pooled pre- (SD_1_) and post-test (SD_2_) standard deviation and pre- and post-test sample size pairs (n) [17]. A majority of studies meeting the inclusion criteria had a sample size of fewer than 20 participants; as such, Hedges’ *g* correction was applied to Cohen’s *d*, as it has been shown to correct for small sample bias [17].

(1) $${\mathrm{Cohen}}^{\prime }s\;d_{av}=\frac{Mdiff}{\frac{SD_{1}+SD_{2}}{2}}$$

(2) $${\mathrm{Hedge}}^{\prime }s\;g_{av}={\mathrm{Cohen}}^{\prime }s\;d_{av}\times \left(1-\frac{3}{4\left(n\right)-9}\right)$$

Values were interpreted as trivial 0.00 < trivial < 0.20, 0.20 ≤ small < 0.60, 0.60 ≤ moderate < 1.20, 1.20 ≤ large < 2.00, 2.00 ≤ very large < 4.00 [18].

## 3. Results

### 3.1. Participant Characteristics

Data for participant and training intervention characteristics are reported as mean ± standard deviation, unless otherwise stated. A total of 14 studies met the inclusion criteria and were included in the review, with a summary of the participant characteristics provided in Table 2. A total of 357 participants were recruited and included in the analysis. Of the 357 total participants, 203 were included in the experimental group, with the remaining 154 participants serving as controls; one study used a crossover design with participants serving as their own controls (*n* = 20). Background variables were provided in all studies except one [19]. Participants took part in a range of team sports including basketball, soccer, handball, and rugby union. Elite junior or academy athletes were recruited in four studies [20,21,22,23]. Athletes from professional or Division I sport organisations were recruited in six studies [19,24,25,26,27,28]. The remainder of studies recruited semi-professional or lower division athletes.

### 3.2. Intervention Characteristics

Training programs lasted from 3 to 10 weeks (8.1 ± 2.6), including 7 to 18 training sessions (13.3 ± 3.1), with the exception of Suarez-Arrones et al. [23], whose intervention included 54 sessions over 27 weeks. Studies utilised a wide range of equipment including free weights typically found in performance settings, inertial flywheel devices or bodyweight-exercise based equipment. The prescribed training volume ranged from one to six sets of 5 to 12 repetitions. One study reported the number of sets (four) but not the number of repetitions [29]. Five studies in total followed the Nordic hamstring exercise protocol (NORD) as described by Mjølsnes et al. [19], which is a 10 week intervention progressively increasing volume from two to three sets of 5 to 12 repetitions utilising the NORD, performed concurrently with soccer specific training. 

The prescription method of exercise intensity used in the experimental groups was quantified in only three studies. These authors prescribed intensity based on percentage of one repetition maximum (1RM) [30], percentage of bodyweight [31] or through a familiarisation protocol [23]. The remaining studies verbally encouraged participants to produce a maximal effort either against a flywheel device of varying inertial resistance [15,20,21,24,28], or during the eccentric phase of bodyweight exercise [19,22,26,29,32]. Compliance to the training intervention was reported in all but three studies [22,24,30]. Compliance values ranged from 70% to 100% (94.5% ± 10.5%). All studies reporting concurrent sport practice in addition to the intervention reported no differences in sport-specific training volume between the experimental and control groups. 

### 3.3. Outcome Measures

#### 3.3.1. Strength

Strength outcomes were assessed in 9 of the 14 studies included in the literature review (Table 3). Five of the nine studies used an isokinetic dynamometer to perform the strength assessment. Training interventions utilised inertial flywheel devices [20,21,23,24,27,28,33], traditional isoinertial equipment [30,31] or exercises performed with bodyweight [19,22,25,26,29]. Mendiguchia et al. [31] used both isoinertial and bodyweight exercises. Studies including the NORD reported effect sizes ranging from trivial (−0.17) [29] to moderate (0.60) [19]. Effect sizes (0.81 to 1.06) were calculated for research involving inertial flywheel devices. Only Cook et al. [30] reported outcome data for strength testing and training with isoinertial equipment. The authors found large (1.22, bench press) to very large (2.16, back squat) effects when eccentric training was compared to traditional training.

#### 3.3.2. Speed

Nine of the fourteen studies reported outcomes measures of speed (Table 4): these measures included velocity [31], top speed [31] and time variables [15,20,22,23,24,26,28,30]. For clarity, positive effect sizes represent a favourable change. Training interventions with flywheel devices reported effect sizes ranging from 0.19 [20] to 0.98 [27]. There were mixed results for NORD training interventions with some unfavourable (−0.07 to −0.60) and favourable (0.20 to 0.81) changes in speed outcomes. EO effects on short sprint (<10 m) times (0.19 to 0.81) [20,22,23,26] were also compared to longer sprint times (>10 m) (−0.60 to 0.98) [20,24,26,27,28].

#### 3.3.3. Power

Explosive movement was measured using a variety of tests (counter-movement jump (CMJ), triple jump, leg press and throwing) in 7 of the 14 studies included in this review (Table 5). CMJ was assessed in six of the studies investigating power. Effect sizes for jump height (CMJ) ranged from small (0.27) to moderate (0.69). Cook et al. [30] combined isoinertial eccentric training with overspeed exercises (1.22). Flywheel studies investigating lower-body power measures reported effect sizes of 0.29 to 1.63. Krommes et al. [26] reported an effect size of 0.27 after a NORD intervention. One study examined upper-body power despite not including upper-body training (−0.13) [28]. 

#### 3.3.4. Change of Direction

Only three studies investigated the effects of eccentric training on change of direction performance (Table 6). The investigation by de Hoyo et al. [21] used force plates to capture kinetic data in crossover and sidestep tasks. Contact time (0.48 to 1.43) and braking time (0.60 to 0.95) both displayed small to moderate effects. Effect size for peak braking force (0.72 to 0.84) and braking impulse (0.53 to 0.92) ranged from small to moderate. Two studies reported [27,33] moderate to large effect sizes (0.93 to 1.46) for changes in Illinois and T-test performance.

## 4. Discussion

The goal of this systematic review was to identify and evaluate the existing literature surrounding EO training interventions and their effects on performance measures in trained team sport athletes. The current evidence is in support of the inclusion of eccentric training in training programs to improve performance measures of strength, speed, power and change of direction. However, inconsistencies exist within the literature with regard to methodologies and variables of interest that need careful consideration before the results can be extrapolated to other athletes or populations. 

The quantification and prescription of EO intensity in well-trained athletes was only reported in four studies focused on performance outcomes [24,25,30,31]. The loading parameters (i.e., load magnitude, repetitions, tempo) were unspecified in several training interventions [19,22,26,29], making it difficult to assess the connection between stimulus and adaptation [34]. Interestingly, Cook et al. [30] were the only authors to prescribe supramaximal training loads (>100% 1RM), even though this method does not require specialised equipment beyond typical barbells and weight plates. The remaining two studies either estimated joint angular velocities [24] or used submaximal loads [31]. Other prescription methods relied on instructing participants to perform one or more phases of the exercise with “maximal effort” [19,22,29,32], but provided no further evidence for EO. Tous-Fajardo et al. [35] demonstrated that the magnitude of eccentric peak force with a flywheel device is largely dictated by the trainee, and that differences in EO exist between those with and without flywheel training experience. Thus, the quantification of neuromuscular and mechanical output data in EO exercises may be necessary to determine the extent of training load and subsequent adaptation.

### 4.1. Strength

Maximum strength, as measured by a single maximal voluntary contraction, is influenced by both neurological and morphological factors that can be influenced through EO [1]. Studies involving trained [36,37,38,39] or untrained participants [40,41] have demonstrated an increase in maximal strength after EO interventions, which may be due to greater neurological contributions and/or type-II muscle fibre hypertrophy. These adaptations are thought to be a result of the high levels of tension developed in the muscle fibres [34], with relatively lower metabolic cost and levels of activation when compared to concentric contractions [42]. These activation patterns may be a result of the distinct molecular and neural characteristics of eccentric contractions [12], and could necessitate specific strategies in order to accurately assess and prescribe eccentric training [43]. A recent review by Douglas et al. [1] highlighted that motor unit recruitment and discharge rates are contributing factors to improvements in eccentric strength following eccentric or heavy resistance training.

The velocity of EO training appears to play an important role in determining subsequent strength adaptation following the intervention. For example, Roig et al. [16] reviewed eccentric and concentric training studies and found that eccentric stimuli produced superior improvements in total strength. However, the effects were greater when the testing and training velocities were matched. The authors concluded that performance outcomes were mode- and velocity-specific. In contrast to these findings, other investigations [41,44] have found that high-velocity EO training (180°·s^−1^) had a greater degree of transfer than slow velocity EO training (30°·s^−1^) during isokinetic testing. Furthermore, one review [45] challenged whether EO provided any additional benefit over traditional training, suggesting that specific populations such as athletes and the untrained may respond differently to EO as a training stimulus depending on baseline strength capabilities.

Studies in the current review reported effect sizes from −0.17 [29] to 1.67 [28] when EO training methodologies were applied to athletes. Training interventions and assessments that were matched on mode of contraction reported effect sizes from 0.19 [25] to 1.67 [28]. Brughelli et al. [29] conducted concentric isokinetic testing following a training intervention that emphasized eccentric contractions. The authors did not speculate whether mode specificity might have played a role in their findings. The only three studies [27,28,30] to report on dynamic (eccentric and concentric) strength tests (1RM leg press, back squat, half squat) demonstrated moderate (0.69) to large (1.67) effects. Although some transfer effect has been noted between contraction modes [6], this phenomenon is inconsistent within the literature [16]. 

Three studies investigating NORD and strength outcomes found small to moderate improvements in trained athletes [19,22,25]. The isokinetic assessments used by these authors matched the mode of contraction used in the training interventions. Ditroilo et al. [46] found that the NORD is capable of producing EMG levels greater than those reported in maximal eccentric isokinetic testing, which supports the NORD as an effective EO exercise. 

The effect and time course of training duration and number of sessions on strength outcomes following EO training is unclear, as both shorter (3 weeks) and longer (10 weeks) interventions and smaller (7) [28] and larger (16) [24] numbers of sessions resulted in improvements. More research is needed to understand the dose–response relationship between EO and strength in trained athletes. With the exception of Brughelli et al. [29], studies investigating strength-based outcomes in athletes appear to be in agreement with the literature that supports EO as a potent stimulus for neuromuscular strength adaptation [1]. 

### 4.2. Speed

The effect of EO training on measures of linear sprint ability are thought to involve numerous components [47]. Briefly, sprint performance consists of several phases, including acceleration and maximal speed, which have distinct kinetic and kinematic features [48]. Acceleration is characterised by extensor action in the hip, knee and ankle, maximal rate of force development over minimal time (RFD), maximal relative strength and higher ground contact time. Maximum-velocity sprinting involves the hip and ankle extensors, RFD and relatively short ground contact time. These features are related to physical qualities such as mechanical stiffness and stretch-shortening cycle (SSC) performance [3,49,50,51,52]). Studies investigating the contribution of mechanical [53] and neuromuscular [36,38,54,55,56] factors have demonstrated improvements as a result of eccentric training.

Several articles identified throughout the literature search were in agreement with the apparent positive effect of EO on sprint performance. Each of the nine studies included in the review reported trivial to moderate improvements in speed measures. Investigations involving short sprints (<10 m) showed trivial to moderate effect sizes. Krommes et al. [26] reported moderate improvements in 5 m (0.81) and 10 m (0.64) sprint times, but moderately slower times in 30 m sprints (−0.60). Cook et al. [30] reported that eccentric training alone did not improve 40 m sprint speed. Mendiguchia et al. [31] described trivial results in 5 m velocity (0.20) and 50 m top speed (−0.07). Additionally, bilateral and unilateral EO training have been reported to increase power and change of direction ability without improving 10 m sprint times [38]. A recent review favoured the use of EO with a flywheel device over traditional resistance training for improving running speed [15]. However, as mentioned previously, controversy exists as to whether certain inertial devices are capable of producing EO [35,57], as force production is in part determined by the experience of the trainee. As previously mentioned, acceleration and maximum-velocity sprinting are distinct physical abilities and may depend on separate performance qualities. Thus, identification and examination of the factors associated with the prescription of EO to enhance specific aspects of sprint performance are necessary.

Contraction velocity appears to be a contributing factor to subsequent adaptations in EO research [1,6,16], with high velocities resulting in greater magnitudes of neuromuscular adaptation. A review by Guilhem et al. [7] stated that isokinetic angular velocity in the literature ranges from 30°·s^−1^ to 210°·s^−1^. Askling et al. [24] were the only authors in the current review to report the joint angular velocity (60°·s^−1^) used in training interventions for speed outcomes, which resulted in a moderate effect (0.73) in trained athletes. Based on previous research investigating high- and low-velocity training [44,58], 60°·s^−1^ may represent a relatively low training velocity. Limited availability of EO literature examining trained populations makes it difficult to draw generalisations; however, it is speculated that distinct sprint qualities may be differentially affected by EO training parameters such as velocity.

### 4.3. Power

The storage and reutilisation of elastic potential energy during the eccentric phase of the SSC is thought to contribute to jump performance [3,59,60,61,62,63]. In the current review, changes in lower-body power performance were measured primarily with jumping variations. These investigations [20,26,27,28,33] revealed small to moderate (0.27 to 0.61) improvements in CMJ performance, while the inclusion of overspeed training protocols resulted in large (1.22) improvements [30]. One reason for the efficacy of EO training in improving power performance may be that relatively fewer motor units are recruited, resulting in more muscle tension, especially at higher velocities [7,41]. The high levels of tension developed in the MTU may lead to adaptive responses in the elastic components of the muscle [34,64]. 

However, investigations using EO have also reported no change in squat jump, counter-movement jump or rate of force development [65]. Additionally, eccentric duration and execution of the correct technique [55,66] may actually decrease measures of velocity in a jump squat. Discrepancy in the effects of EO on power may be a result of individual differences (e.g., technique, anthropometric qualities, contractile and elastic capabilities), which have been shown to influence drop jump and CMJ performance [67]. EO has also been shown to suppress performance qualities for extended periods of time, which could potentially interfere with results dependent on the timing of the post-testing regime [68,69]. Thus, although there seems to be a favourable effect on the expression of power following EO, it is unclear whether the effect sizes related to power production in the literature review are affected by individual recovery profiles or individual differences. 

### 4.4. Change of Direction

Despite recent evidence [9,70,71] on the relationship between eccentric strength and change of direction performance, only three studies in the present review examined any measure of agility. Kinetic data for a novel crossover and sidestep cutting task displayed moderate to large (0.48 to 1.43) changes in the eccentric phase of muscle action following an EO training intervention. Similar effect sizes (0.93 to 1.46) were reported for the Illinois and T-test times in trained athletes [27,33]. Interestingly, neither study reported performing agility-specific training as part of the intervention. This lack of task-specific activities suggests that lower-body flywheel training may transfer to complex skills such as change of direction ability. 

These findings are in agreement with existing literature that has reported improvements in change of direction ability following EO training interventions. Gonzalo-Skok et al. [57] reported substantial improvements in measures of change of direction ability for two EO training programs. The authors reported that although bilateral and unilateral groups improved, differences existed between groups in power outputs and force-vector applications. These results support existing evidence [38,72] for the positive but differential effects of EO on change of direction performance.

## 5. Conclusions

EO appears to be an effective training strategy for athletes and sports practitioners looking to improve measures of strength, speed, power and change of direction. The review highlights evidence suggesting EO can improve performance qualities even in experienced athletes. The exact neurological and morphological mechanisms underlying these changes have been the focus of a growing body of research. Due to a large degree of variation in the existing research, a dose-response relationship for a specific method and its intended adaptation has yet to be determined in trained team sport athletes. Future research should explore the quantification of eccentric ability, the prescription of eccentric training variables and the relationship between EO-induced adaptation and performance qualities.

## Figures and Tables

**Table 1:** Quality assessment for each study included in the analysis.

Author	Inclusion Criteria	Random Allocation	Intervention Defined	Groups Tested for Similarity at Baseline	Control Group	Outcome Variables Defined	Assessments Practically Useful	Duration of Intervention Practically Useful	Between-Group Stats Analysis Appropriate	Point Measures of Variability
Askling et al. (2003)	2	2	2	2	2	2	1	2	2	2
Brughelli et al. (2010)	1	2	1	1	2	2	1	2	2	2
Cook et al. (2013)	0	2	2	1	2	2	2	2	1	2
de Hoyo et al. (2015)	2	0	2	0	2	2	2	2	2	2
de Hoyo et al. (2016)	2	0	2	0	2	2	0	2	2	2
Iga et al. (2012)	0	2	2	2	2	2	1	2	2	2
Ishøi et al. (2018)	2	2	2	0	2	2	1	2	2	2
Krommes et al. (2017)	2	2	2	0	2	2	2	2	0	2
Maroto-Izquierdo et al. (2017)	2	2	2	0	2	2	2	2	2	1
Mendiguchia et al. (2015)	2	2	2	1	2	2	1	2	2	2
Mjølsnes et al. (2004)	0	2	2	0	2	2	1	2	1	2
Sabido et al. (2017)	0	0	2	2	2	2	2	2	2	2
Suarez-Arrones et al. (2018)	2	0	2	0	0	2	2	2	1	1
Sanchez-Sanchez et al. (2019)	0	2	2	0	2	2	2	2	2	2

**Table 2:** Study characteristics for eccentric overload training interventions with male team sport athletes.

Study (Year)	Sample Size	Population	Age (Years)	Height (m)	Body Mass (kg)	Sport	Quality Assessment
Askling et al. (2003)	Exp = 15	Swedish Premier league	24.0 ± 2.6	1.82 ± 0.06	78.0 ± 5.0	Soccer	19
	Con = 15		26.0 ± 3.6	1.81 ± 0.07	77.0 ± 6.0		
Brughelli et al. (2010)	Exp =13	Division 2 Spanish soccer	20.7 ± 1.6	1.80 ± 0.07	73.1 ± 6.0	Soccer	16
	Con = 11		21.5 ± 1.3	1.79 ± 0.07	72.5 ± 7.5		
Cook et al. (2013)	Exp = 5	Semiprofessional rugby union	19.4 ± 0.5	1.85 ± 0.03	93.8 ± 7.0	Rugby Union	16
	Exp = 5		19.8 ± 0.8	1.87 ± 0.05	96.6 ± 9.3		
	Exp = 5		19.6 ± 0.9	1.85 ± 0.04	95.8 ± 7.7		
	Exp = 5		19.8 ± 0.4	1.83 ± 0.05	92.8 ± 6.0		
de Hoyo et al. (2015)	Exp = 18	Division 1 Spanish academy soccer	18.0 ± 1.0	1.78 ± 0.03	70.9 ± 3.9	Soccer	16
	Con = 15		17.0 ± 1.0	1.78 ± 0.01	73.1 ± 2.6		
de Hoyo et al. (2016)	Exp = 17	Division 1 Spanish academy soccer	17.0 ± 1.0	1.78 ± 0.02	71.4 ± 3.9	Soccer	14
	Con = 14						
Iga et al. (2012)	Exp = 10	English Professional League	23.4 ± 3.3	1.77 ± 0.07	78.0 ± 8.2	Soccer	17
	Con = 8		22.3 ± 3.9	1.85 ± 0.09	78.0 ± 11.1		
Ishøi et al. (2018)	Exp = 11	Division 4 Danish academy soccer	19.1 ± 1.8	1.81 ± 0.07	76.2 ± 11.9	Soccer	17
	Con = 14		19.4 ± 2.1	1.81 ± 0.07	77.0 ± 8.7		
Krommes et al. (2017)	Exp = 9	Division 1 Danish professional soccer	23.0 ± 3.9	1.83 ± 0.05	73.1 ± 5.8	Soccer	16
	Con = 10		25.1 ± 4.9	1.81 ± 0.07	77.9 ± 9.9		
Maroto-Izquierdo et al. (2017)	Exp = 15	Division 1 professional handball	19.8 ± 1.0	1.86 ± 0.08	82.3 ± 3.3	Handball	17
	Con = 14		23.8 ± 1.6	1.84 ± 0.01	85.6 ± 3.7		
Mendiguchia et al. (2015)	Exp = 27	Semiprofessional Spanish soccer	22.7 ± 4.8	1.75 ± 0.06	71.6 ± 8.7	Soccer	18
	Con = 24		21.8 ± 2.5	1.77 ± 0.06	71.0 ± 7.7		
Mjølsnes et al. (2004)	Exp = 11	Division 1–4 Danish soccer				Soccer	14
	Exp = 9						
Sabido et al. (2017)	Exp = 11	Division 1 handball	23.9 ± 3.8	1.83 ± 0.07	79.5 ± 7.7	Handball	16
	Con = 10						
Sanchez-Sanchez et al. (2019)	Exp = 12	Regional	22.5 ± 2.2	1.76 ± 0.07	72.6 ± 9.1	Soccer/Basketball	16
	Con = 10						
Suarez-Arrones et al. (2018)	Exp = 14	Serie A Professional	17.5 ± 0.8	1.80 ± 0.06	70.6 ± 5.3	Soccer	12

**Table 3:** Eccentric training intervention characteristics for strength outcomes.

Study (Year)	Weeks	Sessions	Sets × Reps	Equipment	Intensity	Prescription Method	Results
Askling et al. (2003)	10	16	4 × 8	Flywheel	60° s^−1^ or 1.5 s	“Max Effort”	EKFPT (28, 18.9%, *g* = 1.06); CKFPT (20, 15.3, *g* = 0.81)
Brughelli et al. (2010)	4	12	4–5 × ?	Bodyweight	n/a	“Max Effort”	CKFPT (−4, −2%, *g* = −0.17); CKEPT (6, 2.1%, *g* = 0.17)
Cook et al. (2013)	3	12	4 × 5	Isoinertial	80–120% 1RM	%1RM	Bench1RM (*g* = 1.22); Squat1RM (*g* = 0.9)
Iga et al. (2012)	4	9	2–3 × 5–8	Bodyweight	30° s^−1^ or 1 s	“Max Effort”	EKFPT (9 to 20, 7.4% to 20.2%, *g* = 0.19 to 0.54)
Ishøi et al. (2018)	10	12	2–3 × 5–12	Bodyweight	n/a	“Max Effort”	EKFPT (61.7, 19.2%, *g* = 0.94)
Maroto-Izquierdo et al. (2017)	6	15	4 × 7	Flywheel	Two 6.5 kg flywheels with moment inertia of 0.145 kg·m^2^	“Max Effort”	LegPress1RM (31.6, 12.2%, *g* = 0.69)
Mendiguchia et al. (2015)	7	14	1–3 × 2–8	Isoinertial + Bodyweight	5–15 kg or 10–70% BW	Absolute Load + % Bodyweight	CKFPT (16.3 to 18.4, −12.1% to 13.1, *g* = 0.67 to 0.70); EKFPT (31.3 to 42.3, 13.2% to 17.2%, *g* = 0.68 to 0.96)
Mjølsnes et al. (2004)	10	12	2–3 × 5–12	Bodyweight	n/a	“Max Effort”	EKFPT (27, 11.3%, *g* = 0.60)
Sabido et al. (2017)	7	7	2–4 × 8	Flywheel	Flywheel disc with inertia moment of 0.05 kg m^2^	“Max Effort”	HalfSquat1RM (16.5, 14.2%, *g* = 1.67)

*Notes.* Results are reported as (change in mean, % difference, Hedges’ *g*). *Abbreviations.* EKFPT (Eccentric Knee Flexor Peak Torque), CKFPT (Concentric Knee Flexor Peak Torque), CKEPT (Concentric Knee Extensor Peak Torque), Bench1RM (Bench Press 1RM), Squat1RM (Back Squat 1RM), HalfSquat1RM (Half Squat 1RM), and LegPress1RM (Leg Press 1RM).

**Table 4:** Eccentric training intervention characteristics for speed outcomes.

Study (Year)	Weeks	Sessions	Sets × Reps	Equipment	ECC Load/Intensity	Prescription Method	Results
Askling et al. (2003)	10	16	4 × 8	Flywheel	60° s^−1^ or 1.5 s	“Max Effort”	F30 m (−0.08, −2.4%, *g* = 0.73)
Cook et al. (2013)	3	12	4 × 5	Isoinertial	80–120% 1RM	%1RM	Eccentric + Overspeed vs. Traditional 40 m (0.01, *g* = 1.06)
de Hoyo et al. (2015)	10	18	3–6 × 6	Flywheel	Concentric = optimal power output (per inertia = 0.11 kg/m^2^)	“Max Effort”	10 m (−0.02, 1%, *g* = 0.18); F10 m (−0.04, 3.3%, *g* = 0.84); 20 m (−0.04, 1.5%, *g* = 0.30)
Ishøi et al. (2018)	10	12	2–3 × 5–12	Bodyweight	n/a	“Max Effort”	10 m (−0.04, 2.6%, *g* = 0.54)
Krommes et al. (2017)	10	12	2–3 × 5–12	Bodyweight	n/a	“Max Effort”	5 m (−0.09, −10%, *g* = 0.81); 10 m (−0.10, −6%, *g* = 0.64); 30 m (0.10, 2.4%, *g* = −0.60)
Maroto-Izquierdo et al. (2017)	6	15	4 × 7	Flywheel	Two 6.5 kg flywheels with moment inertia of 0.145 kg·m^2^	“Max Effort”	20 m (−0.40, −10.8%, *g* = 0.98)
Mendiguchia et al. (2015)	7	14	1–3 × 2–8	Isoinertial + Bodyweight	5–15 kg or 10–70% BW	Absolute Load + % Bodyweight	v5 m (0.20, 1.0%, *g* = 0.20); v20 m (−0.1, −0.4%, *g* = −0.08); TS (−0.1, −0.3%, *g* = −0.08)
Sabido et al. (2017)	7	7	2–4 × 8	Flywheel	Flywheel disc with inertia moment of 0.05 kg m^2^	“Max Effort”	20 m (−0.08, −2.5%, *g* = 0.82)
Suarez-Arrones et al. (2018)	27	54	1–2 × 5–10	Inertial + Bodyweight	Inertia 0.05 kg/m^2^	Highest power output between two loads during familiarization	10 m (*g* = 0.41); 30 m (*g* = 0.38); 40 m (*g* = 0.31)

*Notes.* Results are reported as (change in mean, % difference, Hedges’ *g*). *Abbreviations.* 5 m (5 m Sprint), 10 m (10 m Sprint), F10 m (Flying 10 m Sprint), 30 m (30 m Sprint) F30 m (Flying 30 m Sprint), v5 m (5 m velocity), v20 m (20 m velocity), and TS (Top Speed velocity).

**Table 5:** Eccentric training intervention characteristics for power outcomes.

Study (Year)	Weeks	Sessions	Sets × Reps	Equipment	ECC Load/Intensity	Prescription Method	Results
Cook et al. (2013)	3	12	4 × 5	Isoinertial	80–120% 1RM	%1RM	Eccentric + Overspeed CMJPP (*g* = 1.22)
de Hoyo et al. (2015)	10	18	3–6 × 6	Flywheel	Concentric = optimal power output (per inertia = 0.11 kg/m^2^)	“Max Effort”	CMJ (2.6, 7.3%, *g* = 0.60)
Krommes et al. (2017)	10	12	2–3 × 5–12	Bodyweight	n/a	“Max Effort”	CMJ (1.15, 2.6%, *g* = 0.27)
Maroto-Izquierdo et al. (2017)	6	15	4 × 7	Flywheel	Two 6.5 kg flywheels with moment inertia of 0.145 kg·m^2^	“Max Effort”	PWR90 (167.5, 21.5%, *g* = 0.71); PWR80 (165.6, 19.7%, *g* = 0.73); PWR70 (113.6, 12.4%, *g* = 0.52); PWR60 (91.5, 10.0%, *g* = 0.41); PWR50 (167.5, 21.5%, *g* = 0.99); CMJ (3.5, 9.8%, *g* = 0.61); SJ (3.3, 9.9%, *g* = 0.54)
Sabido et al. (2017)	7	7	2–4 × 8	Flywheel	Flywheel disc with inertia moment of 0.05 kg·m^2^	“Max Effort”	CMJ (2.4, 6.0%, *g* = 0.47); TJ_R (0.19, 2.9%, *g* = 0.29); TJ_L (0.40, 6.2%, *g* = 0.74)
Sanchez-Sanchez et al. (2019)	5	10	2–3 × 6	Flywheel	Iso-inertial pulley (0.27 kg/ m^2^) and flywheel (0.05 kg/m^2^)	“Max Effort”	CMJ (2.6, 7.4%, *g* = 0.46)
Suarez-Arrones et al. (2018)	27	54	1–2 × 5–10	Inertial + Bodyweight	Inertia 0.05 kg·m^2^	Highest power output between two loads during familiarization	HalfSquat30 (*g* = 0.42); HalfSquat40 (*g* = 0.47); RLHS30 (*g* = 0.48); LLHS30 (*g* = 0.85); RLHS40 (*g* = 1.03); LLHS40 (*g* = 1.63)

*Notes.* Results are reported as (change in mean, % difference, Hedges’ *g*). *Abbreviations.* CMJPP (Counter-movement Jump Peak Power), CMJ (Counter-movement Jump height), HalfSquat30 (Half Squat power at 30 kg), HalfSquat40 (Half Squat power at 40 kg), PWR90 to PWR 50 (Power at 90% to 50% 1RM Leg Press (W)), RLHS30 (Right Leg Half Squat power at 30 kg), LLHS30 (Left Leg Half Squat power at 30 kg), RLHS40 (Right Leg Half Squat power at 40 kg), LLHS40 (Left Leg Half Squat power at 40 kg),TJ_R (Triple Jump Right Leg), and TJ_L (Triple Jump Left Leg).

**Table 6:** Eccentric training intervention characteristics for change of direction outcomes.

Study (year)	Weeks	Sessions	Sets × Reps	Equipment	ECC Load/Intensity	Prescription Method	Results
de Hoyo et al. (2016)	10	18	3–6 × 6	Flywheel	Concentric = optimal power output (per inertia = 0.11 kg/m^2^)	“Max Effort”	BT_crossover (0.01, 16.7%, *g* = 0.60); BT_sidestep (0.01, 16.7%, *g* = 0.95); CT_crossover (0.01, 7.1%, *g* = 0.48); CT_sidestep (0.03, 20.0%, *g* = 1.43); rB_IMP_crossover (0.16, 21.6%, *g* = 0.92); rB_IMP_sidestep (0.13, 13.5%, *g* = 0.53); rPB_crossover (7.4, 29.1%, *g* = 0.72); rPB_sidestep (9.0, 29.7%, *g* = 0.84)
Maroto-Izquierdo et al. (2017)	6	15	4 × 7	Flywheel	Two 6.5 kg flywheels with moment inertia of 0.145 kg·m^2^	“Max Effort”	T-test (0.6, 6.5%, *g* = 1.46)
Sanchez-Sanchez et al. (2019)	5	10	2–3 × 6	Flywheel	Iso-inertial pulley (0.27 kg/ m^2^) and flywheel (0.05 kg/m^2^)	“Max Effort”	Illinois (1.0, 5.6%, *g* = 0.93)

*Notes.* Results are reported as (change in mean, % difference, Hedges’ g). Abbreviations. BT_crossover (Braking Time in crossover cutting), BT_sidestep (Braking Time in sidestep cutting), CT_crossover (Contact Time in crossover cutting), CT_sidestep (Contact Time in sidestep cutting), rB_IMP_crossover (Relative Braking Impulse in crossover cutting), rB_IMP_sidestep (Relative Braking Impulse in sidestep cutting), rPB_crossover (Relative Peak Braking Force in crossover cutting), and rPB_sidestep (Relative Peak Braking Force in sidestep cutting).
